# Trust in Dermatologists Versus Social Media Influencers Among Acne Patients

**DOI:** 10.7759/cureus.83930

**Published:** 2025-05-11

**Authors:** Zulal Inci Bal, Nermin Karaosmanoglu, Berkay Temel, Ozge Mine Orenay

**Affiliations:** 1 Department of Dermatology, Kastamonu Research and Training Hospital, Kastamonu, TUR; 2 Department of Dermatology, Ankara Training and Research Hospital, Ankara, TUR; 3 Department of Dermatology, Gazi University, Ankara, TUR

**Keywords:** acne, dermatologists, effects of social media, influencer, trust in physician

## Abstract

Introduction: Acne vulgaris (AV) is one of the most common skin disorders, particularly affecting young people and adolescents, who are also widespread users of social media.

Objectives: The objectives of this study are to understand why patients seek information about their acne from social media, their perspectives on dermatologists and social media influencers, and how social media sharing affects their views on dermatologists.

Methods: This cross-sectional study included 100 AV patients aged 18-45 who visited the dermatology outpatient clinic of Ankara Training and Research Hospital. Patients were asked to complete a 30-item survey titled "Examination of Acne Patients’ Trust Levels in Social Media Influencers and Dermatologists". The survey was structured to collect data on patients’ social media usage patterns, their sources of information regarding acne (including both dermatologists and social media), and the levels of trust they place in these sources. Descriptive statistics, Mann-Whitney U test, Kruskal-Wallis test, and Chi-square tests were used for analysis, with p<0.05 considered significant.

Results: Most participants (95%) used social media for information. Eighty-one percent of the participants had a high level of trust in their dermatologist, and 97% of participants said that they would trust the dermatologist if the information from the dermatologists and social media influencers were contradictory, and 67% sought acne information from social media influencers. Instagram was the most preferred platform for this purpose (63%). The most common reason for consulting social media was to get information about cosmetic products (36%).

Conclusion: This study found that patients trust dermatologists more than influencers. This makes it important for dermatologists to make informative social media posts on acne. Dermatologists should also reach out to social media influencers and encourage them to share posts by consulting with experts on the topic.

## Introduction

Acne vulgaris (AV) is a chronic inflammatory disease characterized by lesions such as papules, nodules, and pustules [1]. It particularly affects adolescents and young adults [2]. Treatment aims to maximize healing and minimize scarring. Dermo-cosmetic products may be added to the treatment to reduce treatment-related side effects, increase efficacy, and reduce complications such as scarring [3]. In addition to its physical effects, acne has important psychosocial effects. It can reduce patients' confidence and be a reason for challenges in their social relationships [4]. These psychosocial effects can drive patients to seek information from various sources, including social media platforms [5]. Patients especially seek information on social media about cosmetic products, acne treatment options, and the causes of acne [6].

Social media platforms are applications that allow users to create and share content. They began to become widespread in the early 2000s. There is a lot of information about dermatology on social media platforms, including conditions such as acne, psoriasis, and hidradenitis. Social media platforms like Instagram, YouTube, TikTok, and X provide videos and short explanations about acne and its treatment. Social media influencers with millions of followers can influence large numbers of people, especially young people, where acne is more common. However, their lack of training can lead to incorrect information being given to patients [7].

Dermatologists are healthcare professionals specializing in skin disorders. Patients can benefit from professional information and experience by contacting a dermatologist [4].

## Materials and methods

This study included 100 AV patients who presented to the Department of Dermatology at Ankara Training and Research Hospital. The patients were aged between 18 and 45. The local ethics committee approved the study (E-24-8). All participants were informed about the study, and a written consent form was obtained. The study was performed according to the latest version of the "Helsinki Declaration" and "Guidelines for Good Clinical Practice".

Survey questions

A survey titled "Examination of Acne Patients' Trust Levels in Social Media Influencers and Dermatologists" was prepared by reviewing the existing literature and similar studies. The survey consisted of 30 questions in six main sections (see Appendix A). The sections and content of the survey are presented in detail below:

General Patient Information

In this section, demographic information such as patients' age, gender, education status, and severity of acne was collected. When researching education status, primary school, high school, and university options are presented. Primary education in Türkiye lasts eight years with no clear distinction between primary and secondary school levels, followed by four years of high school education. Therefore, secondary school education does not exist as a separate stage in the curriculum. When researching, the severity of acne was not assessed using a validated scoring system. Instead, it was classified into mild, moderate, and severe categories based on the clinicians’ overall clinical evaluation. This information was used to understand the diversity of the study group and to examine the effect of demographic factors in the analyses.

Consultation Frequency for Information

Participants were asked how often they consulted dermatologists or social media influencers regarding acne treatment. This section determined which information source patients preferred and how often during their treatment process.

Top Preferred Social Media Platform and Trend Topics Researched

Participants were asked which social media platform they preferred and which topics they investigated on these social media platforms.

Trust in Dermatologists

Participants were asked to indicate their trust levels in dermatologists. The level of trust was assessed using a scoring system from 1 to 5. The factors affecting the level of trust in social media influencers were noted. This section also aimed to analyze the effects of advice received from dermatologists.

Trust in Social Media Influencers

This section evaluated the participants’ trust and satisfaction levels with the advice they received from social media influencers. The level of trust was assessed using a scoring system from 1 to 5. The factors affecting the level of trust in social media influencers were noted. This section aimed to analyze the effects of advice received from social media influencers.

The Frequency of Usage and Satisfaction Levels of the Products Recommended by Dermatologists or Social Media Influencers

The patients were asked for the frequency of usage as "always", "frequently", "rarely", and "never". The level of satisfaction was scored as "very satisfied", "satisfied", "neutral", and "unsatisfied".

The survey included multiple-choice questions, a rating system, and open-ended questions where participants could freely express their opinions. In this way, comprehensive and qualified data were obtained regarding the participants' sources of information, confidence levels, and experiences. At the end of the survey, a score of 4 or 5 was considered as "definite trust" and "high satisfaction", while scores between 1 and 3 were considered as "low trust" and "low satisfaction".

Statistical analysis

While evaluating the findings obtained in the study, the IBM SPSS Statistics for Windows, Version 22 (Released 2015; IBM Corp., Armonk, New York, United States) was used for statistical analyses. While evaluating the study data, in addition to descriptive statistical methods (minimum, maximum, mean, standard deviation, median, frequency), the Kruskal-Wallis test was used for comparisons of parameters not showing normal distribution between groups in comparisons of quantitative data, and Mann-Whitney U test was used for comparisons between two groups. In comparisons of qualitative data, the chi-square test, Fisher’s exact chi-square test, Fisher-Freeman-Halton exact chi-square test, and Yates continuity correction were used. Significance was assessed at a p<0.05 level.

## Results

Of the 100 patients in this study, 75 (75%) were women and 25 (25%) were men. The mean age was 20.93±3.17 years. The demographic features of the patients are shown in Table 1.

**Table 1:** The demographic data of the participants

Demographic Features		n	%
Gender	Male	25	25
	Female	75	75
Education level	Primary school	5	5
	High school	36	36
	University	59	59
Severity of acne	Mild	16	16
	Moderate	51	51
	Sever	29	29
	Very severe	4	4
Using social media as a source of information	Yes	95	95
	No	5	5
Social media platforms used	Instagram	92	92
	Twitter	41	41
	Facebook	6	6
	TikTok	26	26
	YouTube	14	14
	Not using	5	5

**Figure 1:**
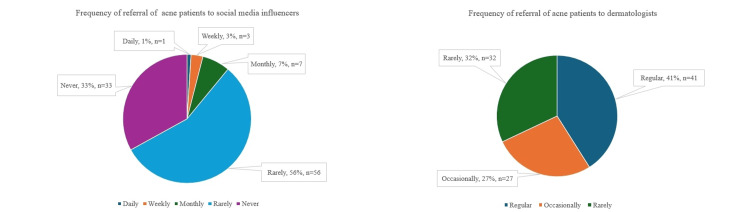
Frequency of acne patient referrals from social media influencers versus dermatologists

Dermatologists were the first choice for acne treatment recommendations for 97% of patients. For 10% of the patients, the first preferred source was both a dermatologist and a social media influencer. The frequency of referrals of patients to dermatologists and social media influencers regarding acne is shown in Figure 1, and 67% of all of the participants revealed that they searched for their questions on social media.

**Figure 2:**
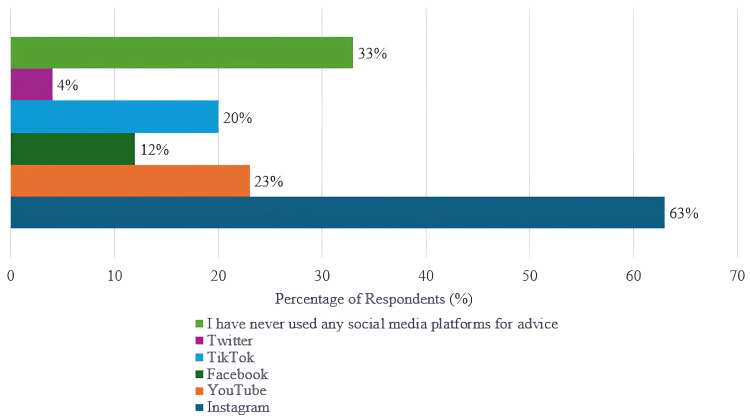
Frequency of social media platforms used by patients for acne-related product and treatment recommendations

**Figure 3:**
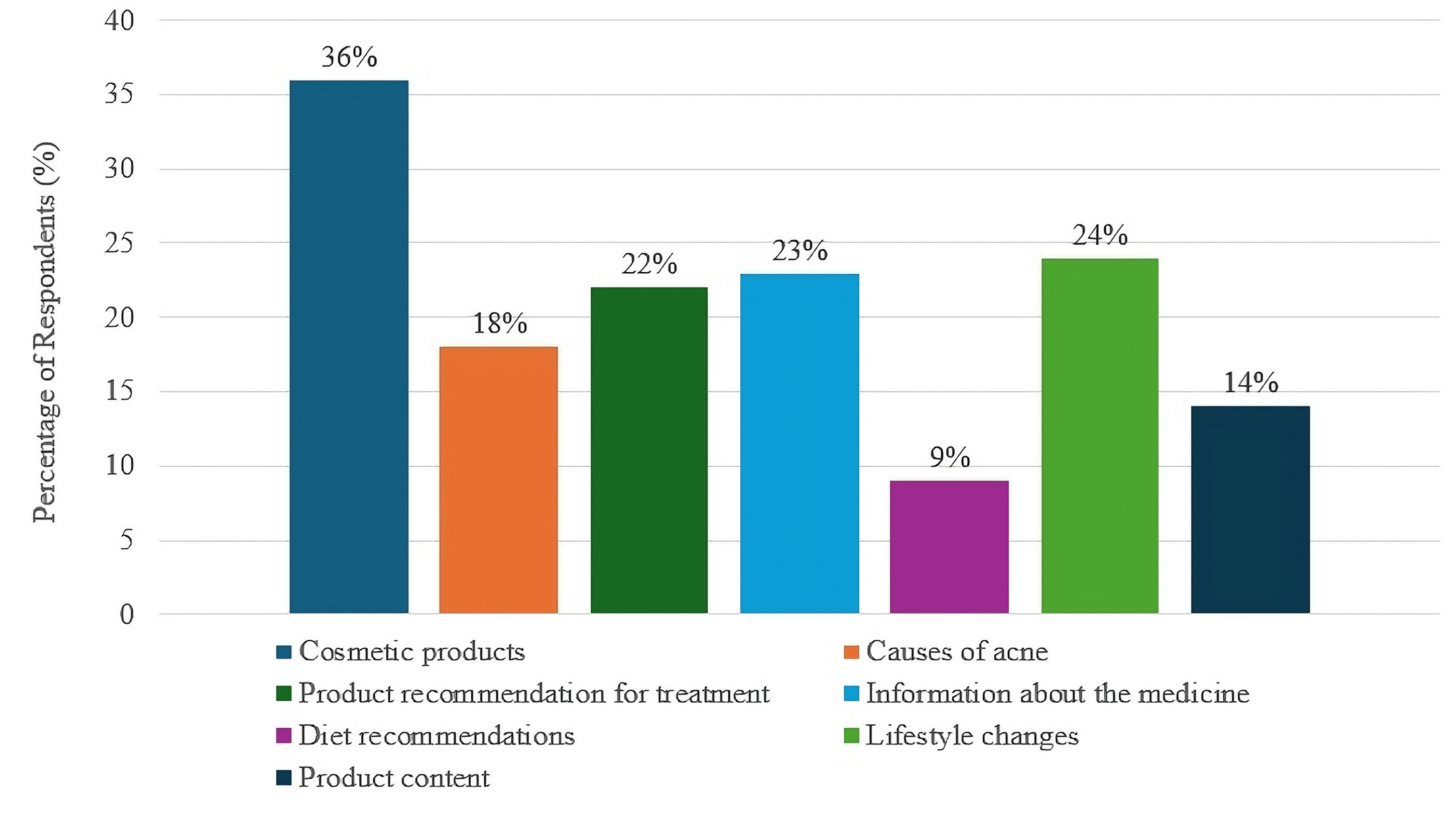
Reasons patients turn to social media for acne-related information

While Instagram was the most preferred platform (Figure 2), "cosmetic products used for acne" was the most trending topic. On the other hand, the least trending topic was detected to be "dietary recommendations for acne" (Figure 3).

A statistically significant association was found between gender, education level, and the likelihood of consulting social media influencers for acne (Table 2).

**Table 2:** Evaluation of factors associated with seeking treatment recommendations from social media influencers for acne ^1^ Yates continuity correction; ^2^ Fisher-Freeman-Halton exact test; ^3^ Fisher’s exact test; * p<0.05 (statistically significant)

Variable		Yes	No	P-value
		n (%)	n (%)	
Gender	Male	10 (40)	15 (60)	^1^0.002*
	Female	57 (76)	18 (24)	
Education level	Primary school	2 (40)	3 (60)	^2^0.004*
	High school	18 (50)	18 (50)	
	University	47 (79.7)	12 (20.3)	
Severity of acne	Mild	13 (81.3)	3 (18.8)	^2^0.494
	Moderate	34 (66.7)	17 (33.3)	
	Severe	17 (58.6)	12 (41.4)	
	Very severe	3 (75)	1 (25)	
Who do you first turn to for information and advice when you have questions about acne?	Dermatologists	57 (63.3)	33 (36.7)	^3^0.028*
	Both equally	10 (100)	0 (0)	
How satisfied are you with the medical treatment and advice you have received for acne?	Very dissatisfied	2 (50)	2 (50)	^2^0.219
	Dissatisfied	11 (91.7)	1 (8.3)	
	Neutral	26 (63.4)	15 (36.6)	
	Satisfied	20 (60.6)	13 (39.4)	
	Very satisfied	8 (80)	2 (20)	

The frequency of consulting social media influencers for acne-related content was significantly higher among Instagram (58.9%) and TikTok users (16.7% weekly, 69.2% rarely) compared to non-users (Instagram: p=0.022; TikTok: p=0.015; p<0.05). Additionally, YouTube users were significantly more likely than non-users to seek acne-related information from social media influencers (p=0.017) (Table 3).

**Table 3:** Evaluation of the frequency of referring to social media influencers’ posts for acne based on personal information ^1 ^Fisher-Freeman-Halton Exact test; * p<0.05

Platform		Frequency of referring to social media influencers’ posts for acne					P-value
		Daily	Weekly	Monthly	Rarely	Never	
		n (%)	n (%)	n (%)	n (%)	n (%)	
Instagram	Yes	1 (1.1)	3 (3.3)	7 (7.6)	55 (59.8)	26 (28.3)	^1^0.022*
	No	0 (0)	0 (0)	0 (0)	1 (12.5)	7 (87.5)	
Twitter	Yes	1 (2.4)	2 (4.9)	3 (7.3)	26 (63.4)	9 (22)	^1^0.184
	No	0 (0)	1 (1.7)	4 (6.8)	30 (50.8)	24 (40.7)	
Facebook	Yes	0 (0)	1 (16.7)	0 (0)	4 (66.7)	1 (16.7)	^1^0.309
	No	1 (1.1)	2 (2.1)	7 (7.4)	52 (55.3)	32 (34)	
Tiktok	Yes	0 (0)	2 (7.7)	3 (11.5)	18 (69.2)	3 (11.5)	^1^0.015*
	No	1 (1.4)	1 (1.4)	4 (5.4)	38 (51.4)	30 (40.5)	
Youtube	Yes	0 (0)	2 (14.3)	2 (14.3)	9 (64.3)	1 (7.1)	^1^0.017*
	No	1 (1.2)	1 (1.2)	5 (5.8)	47 (54.7)	32 (37.2)	

**Figure 4:**
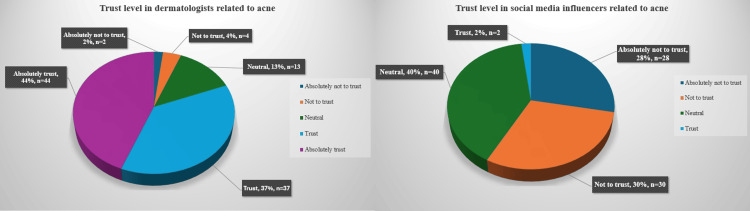
Level of trust in dermatologists and social media influencers regarding acne

The majority of patients (81%) indicated their level of confidence in dermatologists as "definite trust" (scores 4 or 5). For social media influencers, this rate was just 2% (Figure 4).

Gender and education level did not seem to have any statistically significant effect on the level of confidence of patients in dermatologists and social media influencers (Table 4).

**Table 4:** Evaluation of trust levels in dermatologists and social media influencers regarding acne, based on general information ^1 ^Mann-Whitney U Test; ^2 ^Kruskal-Wallis test

Variable		Level of trust in dermatologists regarding acne, Mean±SD (Median)	P-value	Level of trust in social media influencers regarding acne, Mean±SD (Median)	P-value
Gender	Male	3.96 ± 0.98 (4)	^1^0.160	2.16 ± 0.94 (2)	^1^0.993
	Female	4.24 ± 0.93 (4)		2.16 ± 0.84 (2)	
Education level	Primary education	4.00 ± 1.73 (5)	^2^0.486	1.60 ± 0.89 (1)	^2^0.133
	High school	3.97 ± 1.11 (4)		2.03 ± 0.84 (2)	
	University	4.31 ± 0.73 (4)		2.29 ± 0.85 (2)	

**Figure 5:**
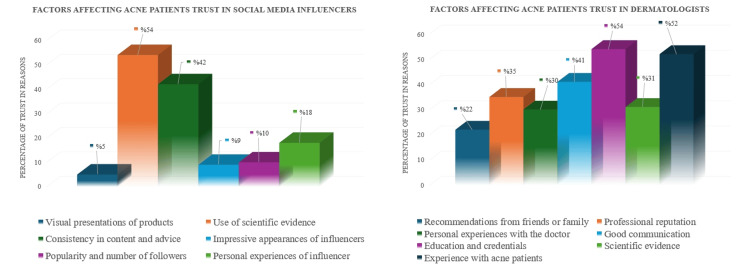
Factors affecting acne patients' trust in social media influencers and dermatologists

The most common factor affecting patients’ confidence in dermatologists was "education and credentials". For social media influencers, this factor was "the use of scientific evidence" (Figure 5).

It was determined that factors such as male gender and being a primary school graduate affect the level of trust in dermatologists and create a tendency to seek solutions on social media platforms (Table 5).

**Table 5:** Evaluation of the impact of social media content on patients’ trust in dermatologists ^1 ^Fisher-Freeman-Halton Exact test;^ 2 ^Fisher’s exact test; * p<0.05

Variable		Did the social media content you watched have any effect on your trust in doctors?			P-value
		No	Yes	I didn't watch	
		n (%)	n (%)	n (%)	
Gender	Male	10 (40)	1 (4)	14 (56)	^1^0.011*
	Female	53 (70.7)	4 (5.3)	18 (24)	
Education Level	Primary school	1 (20)	1 (20)	3 (60)	^2^0.001*
	High school	18 (50)	0 (0)	18 (50)	
	University	44 (74.6)	4 (6.8)	11 (18.6)	

Fifty-five percent of patients reported that they "always" used products recommended by dermatologists, 37% reported that they used them "frequently", and 7% reported that they used them "rarely". Only 1% of patients indicated that they "never" used these products. A small percentage of patients, just 6%, indicated their satisfaction with the advice they received from social media influencers as "very satisfied" or "satisfied". Twenty-three percent of patients said that their acne improved as a result of advice they received from social media influencers. However, side effects such as erythema and increased acne were reported in 7% of patients.

Patients who received acne advice from both social media influencers and dermatologists reported greater improvement in their acne than those who received advice from only dermatologists (p:0.033; p<0.05) (Table 6).

**Table 6:** Evaluations regarding the decrease in acne after receiving recommendations ^1 ^Chi-square test; ^2 ^Fisher-Freeman-Halton exact test; * p<0.05

Variable		Did the acne decrease after following the recommendations?			P-value
		Yes	No	I did not receive any recommendations	
		n (%)	n (%)	n (%)	
Level of trust in social media influencers regarding acne	Absolutely distrust	2 (7.1)	11 (39.3)	15 (53.6)	^1^0.031*
	Distrust	7 (23.3)	13 (43.3)	10 (33.3)	
	Neutral	14 (35)	18 (45)	8 (20)	
	Trust	0 (0)	2 (100)	0 (0)	
Who was consulted for information and advice regarding acne?	Dermatologists	19 (21.1)	38 (42.2)	33 (36.7)	^2^0.033*
	Both are equal	4 (40)	6 (60)	0 (0)	
The situation of seeking treatment recommendations from social media influencers for acne	Yes	23 (34.3)	44 (65.7)	0 (0)	^2^0.001*
	No	0 (0)	0 (0)	33 (100)	

The study also found that women and university graduates were more likely to consult dermatologists before using products recommended by social media influencers (Table 7).

**Table 7:** Evaluations of the level of consulting a dermatologist before using recommendations obtained from social media ^1 ^Fisher-Freeman-Halton exact test; ^2 ^Chi-square test; * p<0.05

Variable		Always	Frequently	Rarely	I never consult	P-value
		n (%)	n (%)	n (%)	n (%)	
Gender	Male	0 (0)	2 (8)	1 (4)	0 (0)	^1^0.001*
	Female	12 (16)	16 (21.3)	16 (21.3)	6 (8)	
Education Level	Primary school	2 (40)	0 (0)	0 (0)	0 (0)	^2^0.009*
	High school	0 (0)	4 (11.1)	5 (13.9)	2 (5.6)	
	University	10 (16.9)	14 (23.7)	12 (20.3)	4 (6.8)	

Patients were less satisfied with the recommendations they received from social media regarding "lifestyle changes" and "diet" than with the recommendations regarding "cosmetic products" and "acne treatment drugs" (Table 8).

**Table 8:** Evaluation of the level of satisfaction with advice received from social media influencers on acne ^1 ^Fisher-Freeman-Halton exact test; * p<0.05

Variable		How satisfied are you with the advice you received from social media influencers for your acne?					P-value
		Very dissatisfied	Dissatisfied	Normal	Satisfied	Not seeking advice	
		n (%)	n (%)	n (%)	n (%)	n (%)	
Causes of acne	Yes	1 (5.6)	5 (27.8)	10 (55.6)	2 (11.1)	0 (0)	^1^0.001*
	No	9 (11)	10 (12.2)	28 (34.1)	2 (2.4)	33 (40.2)	
Product recommendation for treatment	Yes	2 (9.1)	7 (31.8)	12 (54.5)	1 (4.5)	0 (0)	^1^0.001*
	No	8 (10.3)	8 (10.3)	26 (33.3)	3 (3.8)	33 (42.3)	
Product ingredients	Yes	3 (21.4)	3 (21.4)	5 (35.7)	3 (21.4)	0 (0)	^1^0.001*
	No	7 (8.1)	12 (14)	33 (38.4)	1 (1.2)	33 (38.4)	
Information or side effects regarding medication used for acne treatment	Yes	3 (13)	3 (13)	15 (65.2)	2 (8.7)	0 (0)	^1^0.001*
	No	7 (9.1)	12 (15.6)	23 (29.9)	2 (2.6)	33 (42.9)	
Dietary modification	Yes	2 (22.2)	2 (22.2)	4 (44.4)	1 (11.1)	0 (0)	^1^0.075
	No	8 (8.8)	13 (14.3)	34 (37.4)	3 (3.3)	33 (36.3)	
Lifestyle modification	Yes	5 (20.8)	5 (20.8)	13 (54.2)	1 (4.2)	0 (0)	^1^0.001*
	No	5 (6.6)	10 (13.2)	25 (32.9)	3 (3.9)	33 (43.4)	
Cosmetic products	Yes	5 (13.9)	8 (22.2)	20 (55.6)	3 (8.3)	0 (0)	^1^0.001*
	No	5 (7.8)	7 (10.9)	18 (28.1)	1 (1.6)	33 (51.6)	

In case of disagreement between dermatologists and social media influencers, almost all patients revealed that they would trust dermatologists (97%). When asked if it was helpful for them to have dermatologists share informative content on social media, 91% of patients answered “yes”. There was a positive correlation between female gender and finding this situation helpful. In addition, a significant difference was observed in the perceived usefulness of dermatologists sharing informative videos on social media across different levels of daily average social media usage (p=0.008; p<0.05). As daily usage time increased, the proportion of participants who found such content beneficial also increased (Table 9).

**Table 9:** Evaluation of the perception of the usefulness of dermatologists publishing informative videos on social media platforms ^1 ^Fisher’s exact test; ^2^ Fisher-Freeman-Halton exact test; * p<0.05

Variable		Finding it beneficial for dermatology doctors to publish informative videos on social media platforms		P-value
		Yes	No	
		n (%)	n (%)	
Gender	Male	20 (80)	5 (20)	^1^0.041*
	Female	71 (94.7)	4 (5.3)	
Education Level	Primary school	5 (100)	0 (0)	^2^0.192
	High school	30 (83.3)	6 (16.7)	
	University	56 (94.9)	3 (5.1)	
Using social media as an information source	Yes	86 (90.5)	9 (9.5)	^1^1.000
	No	5 (100)	0 (0)	
Average daily social media usage time	Less than 30 minutes	1 (25)	3 (75)	^2^0.008*
	30 minutes to 1 hour	4 (80)	1 (20)	
	1 to 2 hours	21 (95.5)	1 (4.5)	
	2 to 3 hours	17 (94.4)	1 (5.6)	
	3 to 4 hours	23 (92)	2 (8)	
	4 to 5 hours	15 (100)	0 (0)	
	More than 5 hours	5 (83.3)	1 (16.7)	
	I don’t use it	5 (100)	0 (0)	

## Discussion

AV is a chronic inflammatory disease that usually affects young people and women [1]. It significantly impairs patients’ quality of life, as demonstrated in multiple studies. For instance, a study by Hanisah et al. (2009) found that acne had a substantial negative impact on the quality of life among adolescents in Malaysia [8]. Social media platforms are sources where information can be shared on different topics. Dermatology-related topics are quite popular on social media platforms. There are a lot of posts on social media, especially about acne. Women notice facial lesions more than men and care more about their appearance [9]. Due to hormonal changes, they could have acne lesions more frequently, especially during the premenstrual period. As expected, in this study, the frequency of female patients was higher (75%). In a review that examined 14 studies, it was found that 16- to 20-year-old patients were the most commonly ones to be affected by acne [10]. In this study, the median age of patients was 20.93±3.17.

Factors such as age, gender, and socioeconomic status may affect patients' search for health services [11]. In two independent studies, it was determined that women and university graduates tend to seek recommendations about acne on social media more frequently [12,13]. In this study, women and university graduates were found to consult social media influencers about acne more frequently than men and primary school graduates. This may be explained by women’s greater health awareness and more active use of social media. Additionally, the societal emphasis on women’s physical appearance, the cosmetics industry’s focus on female consumers, and women’s greater willingness to share their skin concerns may increase their exposure to acne-related content [14-16]. University graduates may have access to more information, evaluate content on social media more critically, and have a greater desire to learn. These factors may explain why these patients search for more acne-related information on social media compared to people with other education levels.

Social media provides easy and fast access to information. Patients can turn to social media for quick solutions and practical advice. Additionally, dermatologists and influencers can offer different perspectives and experiences. Patients may want to see the experiences of other patients as well as professionals. There is a lot of information on social media, including advice on acne-related products, diet, and lifestyle changes. In a study, it was found that some social media influencers shared informative content about medications used in acne treatment [17]. Another study found that patients often use social media to search for over-the-counter acne treatments [18]. Cosmetics are products such as sunscreens, moisturizers, and facial cleansers. In a study conducted in 2019, it was reported that Dr. Sandra Lee, who shares cosmetic content, was the most-followed dermatologist on Instagram and YouTube [19]. This study found that acne patients most commonly use social media to get cosmetic product recommendations. These were followed by lifestyle changes, information about medications used in treatment, and the causes of acne. When all the results are evaluated together, it can be considered that it would be important for dermatologists who are experts in this field to provide more information about acne, especially cosmetic products, on social media in order to inform patients correctly.

Patients can use various social media platforms to learn about acne. Instagram and YouTube were found to be the most commonly used social media platforms by acne patients in a study by Kaliyadan et al. [20]. In this study, it was also found that the platforms most commonly used by acne patients were Instagram, YouTube, and Facebook. On these platforms, patients can find visual posts about treatment methods and results. They can read other users' experiences regarding the treatment process. They can also see product reviews and hands-on content on skincare routines. Social media influencers may share detailed videos about acne. These results suggest that patients seeking information about acne view social media platforms as an important source of information and that the influencers found on these platforms have a widespread effect on patients. Thus, it can be concluded that the search for health-related information has changed with the influence of digitalization and social media.

In this study, the frequency of consulting social media influencers for acne-related content was found to be significantly higher among Instagram and TikTok users compared to non-users (Instagram: p=0.022; TikTok: p=0.015; p<0.05). TikTok and Instagram are platforms that prioritize short, visually engaging content tailored to users’ personal interests through algorithmic curation. This structure increases both the visibility of influencer posts and the perceived credibility of the content. Particularly among young adult acne patients, such platforms can lead to frequent exposure to unverified skincare advice. These types of content may influence patients’ treatment preferences or delay their decision to seek professional medical help. Therefore, it is crucial for dermatologists to increase the dissemination of accurate and reliable information in alignment with the dynamics of these widely used platforms.

Schoenberg et al. researched the most investigated dermatological topics on social media. They found that acne was in the fourth place [21]. Another study found that acne is a topic that patients seek information about on social media [10]. Consistent with the literature, this study found that 67% of patients sought acne information from social media. This situation may have negative effects as social media influencers do not have professional medical knowledge. In their study, Reddy et al. found that there was much false information about acne on Instagram [17]. This may expose patients to misinformation. Some social media influencers may collaborate with brands to promote cosmetic products. A study found that social media influencers use these platforms to promote their own products [22]. This may result in subjective recommendations. Additionally, the flawless-looking skin of influencers can have a negative impact on patients. This situation can lead to a decrease in self-confidence and even depression. Therefore, it may be recommended that an advisory board of health professionals review such posts to prevent misinformation originating from social media.

Many factors affect acne patients’ trust in dermatologists. This study presents an important finding that is not found in other studies in the literature. When the patients participating in the study were asked about “factors affecting trust in dermatologists”, the most common response was education and credentials; and when asked “factors affecting trust in social media influencers”, the most common response was the use of scientific evidence. It was determined that participants who trusted dermatologists were more likely to attach importance to education, credentials, and scientific evidence. In addition, the most common reason participants trusted products recommended by dermatologists was that they were recommended by professionals. Additionally, this result suggests that AV patients, who are in the younger age group that frequently uses social media, tend to give more importance to professional recommendations about acne. This is an important indicator for understanding how young people evaluate information sources and reliability when making health-related decisions.

In this study, it was determined that the level of trust in dermatologists was not affected in total in terms of gender. On the other hand, it was found that the content watched on social media by male patients, primary school graduates, and patients who turned to YouTube for acne-related suggestions had a higher rate of affecting their trust in dermatologists. Although the tendency to care about appearance and the tendency to be "better looking" has increased in men in recent years, it is still widely thought in some segments that giving too much importance to appearance is characteristic specific for women. Consequently, male patients may delay or postpone their visit to a dermatologist for skin problems. Social media content may provide male patients with the opportunity to secretly obtain information and research treatment options. Thus, male patients may be more influenced by social media content. Being a primary school graduate may also lead to having less knowledge of dermatological issues. In this case, social media content may be more understandable or accessible to them than to dermatologists. These patients may have a more critical attitude towards traditional medical approaches or doctor visits due to certain social, cultural, or economic factors. This may cause them to rely more on alternative treatment methods or information sources.

In this study, it was found that patients seeking information about acne on social media were less satisfied with the recommendations they received regarding lifestyle changes, dietary changes, and cosmetic products than they were with the information they received about medications used for acne treatment. This may mean that there is not enough satisfactory information on social media about factors such as cosmetic products, lifestyle changes, and dietary changes that are necessary to support acne treatment. Therefore, dermatologists can contribute to patients' knowledge of acne by providing effective and reliable information on social media, especially in these areas. However, Trepanowski and Grant-Kels suggested that dermatologists should avoid promoting products on social media due to potential ethical concerns, such as dermatologists receiving payment for promoting products on social media [23]. By taking adequate precautions regarding ethical problems, educational posts can be made on social media, and a wide audience can be reached by dermatologists in this way.

In this study, a difference was observed in the perceived usefulness of dermatologists sharing informative videos on social media depending on participants’ average daily social media usage time. As daily usage time increased, the proportion of participants who found such content beneficial also increased. These findings suggest that individuals who spend more time on social media may be more receptive to professional medical content, such as dermatologist-created videos. This highlights the importance of delivering evidence-based information in a format and medium that aligns with users’ digital habits.

The majority of patients in this study (91%) thought that it could be beneficial if the dermatologists post informative content on social media. There are studies in the literature that support this data. Szeto et al. have suggested that dermatologists could reach wider audiences by using more social media platforms [24]. Another study found that patients want to find more correct information on social media and want dermatologists to provide recommendations and create blogs on social media [12]. These results indicate that social media is becoming increasingly more important in dermatology. When these results are evaluated together, it can be thought that patients want to receive professional advice and expect dermatologists to be more active on these platforms.

Limitations

There are some limitations of the present study. Firstly, patients between the ages of 12 and 18 who actively use social media were not included. Secondly, it included a relatively moderate number, and future studies including a larger number of patients are needed. Finally, the survey questions were subjective, and participants answered them based on their own experiences.

## Conclusions

In conclusion, this study has shown that although the majority of AV patients seek various acne-related suggestions on social media platforms, they tend to place more trust in dermatologists. This finding highlights the importance of maintaining a strong and reliable medical presence on digital platforms. Improving the accessibility and visibility of evidence-based dermatological information on social media can support more informed patient decision-making and encourage greater adherence to professional medical advice.

## Appendices

Appendix A 

**Table 10:** Comparison of acne patients’ trust in social media influencers and dermatologists

Section	Question	Answer Options
1. General Information	1.1. Name-Surname	Open-ended (X** Y**)
	1.2. Age/Gender	Open-ended
	1.3. Education Level	Open-ended
	1.4. Duration of Acne	Open-ended
	1.5. Severity of Acne	Mild/Moderate/Severe/Very Severe
	1.6. Do you use social media applications as a source of information?	Yes/No
	1.7. Which social media platforms do you use?	Instagram, Twitter, Facebook, TikTok, YouTube
	1.8. How many hours a day do you spend on social media on average?	Less than 30 minutes/30 min-1 hour/1-2 hours/2-3 hours/3-4 hours/4-5 hours/More than 5 hours/I do not use social media
2. Trust in Information Sources	2.1. Level of trust in dermatologists (1-5)	1 (Absolutely Insecure)/2 (Insecurity)/3 (Neutral)/4 (Trust)/5 (Absolutely Trust)
	2.2. Level of trust in social media influencers (1-5)	1 (Absolutely Insecure)/2 (Insecurity)/3 (Neutral)/4 (Trust)/5 (Absolutely Trust)
	2.3. How often do you see a dermatologist for your acne?	Regularly/Occasionally/Rarely/Once/Never/Prefer not to answer
	2.4. How often do you refer to social media influencers' posts for your acne?	Daily/Weekly/Monthly/Rarely/Never/I haven’t applied yet but I plan to/Prefer not to answer
3. Factors Affecting Trust	3.1. What factors affect your trust in dermatologists?	Education and credentials, Experience with acne patients, Professional reputation, Personal experiences, Recommendations from friends or family, Scientific evidence, Good communication
	3.2. What factors affect your trust in social media influencers?	Personal experiences, Appealing appearance, Consistency in content and advice, Visual presentation of products, Use of scientific evidence, Popularity and number of followers
4. Searching for Acne Information	4.1. Who do you turn to first for information and advice about acne?	Dermatologists/Social media influencers/Both equally/I do not seek information or advice
5. The Role of Dermatologists	5.1. Have you consulted a dermatologist for your acne?	Yes/No
	5.2. Satisfaction with medical treatment and advice	Very dissatisfied/Dissatisfied/Normal/Pleased/Very Pleased
	5.3. How often do you use acne treatment products recommended by your dermatologist?	Always/Often/Rarely/Never
	5.4. Why do you trust the products recommended by your dermatologist?	Professional advice, Scientifically based recommendations, Obtaining products from pharmacies, Other (Specify)
6. The Role of Social Media Influencers	6.1. Have you sought treatment advice for your acne from social media influencers?	Yes/No
	6.2. From which platforms did you receive acne-related product or treatment recommendations?	Instagram, Twitter, YouTube, TikTok, Facebook, I never got recommendations, Other (Specify)
	6.3. What acne-related advice have you sought from social media?	Causes of acne, Product recommendations, Product ingredients, Medication side effects, Diet change, Lifestyle changes, Cosmetic products, I don’t seek acne advice on social media
	6.4. Satisfaction with advice received from social media influencers	Very dissatisfied/Dissatisfied/Normal/Pleased/Very Pleased/I did not get advice from social media for acne
	6.5. Did your acne improve after following the recommendations?	Yes/No/I didn’t get recommendations
	6.6. Did you experience any side effects from the recommendations?	Yes (Specify)/No/I didn’t get recommendations
	6.7. Do you think it would be beneficial for dermatologists to post informative videos on social media?	Yes/No
	6.8. Did the social media content you watched affect your trust in doctors?	No/Yes (Specify)/I didn’t watch acne-related content
	6.9. If information from social media contradicts a dermatologist’s advice, which do you trust more?	Social media content/Dermatologist/Depends on the situation (Specify)
	6.10. How often do you consult health professionals when using products recommended by social media influencers?	Always/Often/Rarely/Never/I do not use products recommended by social media influencers
7. Final Thoughts	7.1. Is there anything specific that impacts your trust in medical doctors or influencers regarding acne?	Open-ended
